# Claudins—Promising Biomarkers for Selected Gastrointestinal (GI) Malignancies?

**DOI:** 10.3390/cancers16010152

**Published:** 2023-12-28

**Authors:** Marta Łukaszewicz-Zając, Barbara Mroczko

**Affiliations:** 1 Department of Biochemical Diagnostics, Medical University, Waszyngtona 15 a, 15-269 Bialystok, Poland; barbara.mroczko@umb.edu.pl; 2 Department of Neurodegeneration Diagnostics, Medical University, 15-269 Bialystok, Poland

**Keywords:** biomarker, claudins, gastrointestinal tumours

## Abstract

**Simple Summary:**

Claudins (CLDNs) belong to a family of tetraspan transmembrane proteins of the tight junctions (TJs) which form the paracellular barrier that regulates the flow of molecules in the intercellular space between the cells of the epithelium. Evidence of altered claudin levels in various human malignancies, including GI cancers, has been reported. GI cancers are commonly diagnosed malignancies worldwide and their incidence is still on the increase, particularly in Western countries. Therefore, there is an urgent need for more investigations into early biomarkers of these malignant diseases. In this review, we present the involvement of selected CLDNs in the pathogenesis of the most common GI malignancies. Moreover, the usefulness of these proteins in the diagnosis of GI cancers and patient prognosis has also been evaluated. Our review indicates that selected CLDNs, particularly CLDN1, 2, 4, 7, and 18, play a significant role in the development of GI tumours and in patient prognosis. In addition, selected CLDNs may be of value in the design of therapeutic strategies for the treatment of recurrent tumours.

**Abstract:**

Despite recent improvements in diagnostic ability and treatment strategies for patients with neoplastic disease, gastrointestinal (GI) cancers, such as colorectal, gastric, pancreatic, and oesophageal cancers, are still common malignancies and the leading cause of cancer deaths worldwide, with a high frequency of recurrence and metastasis as well as poor patient prognosis. There is a link between the secretion of proteolytic enzymes that degrade the extracellular matrix and the pathogenesis of GI tumours. Recent findings have focused on the potential significance of selected claudins (CLDNs) in the pathogenesis and prognosis of GI cancers. Tight junctions (TJs) have been proven to play an important role in maintaining cell polarity and permeability. A number of authors have recently revealed that TJ proteins, particularly selected CLDNs, are related to inflammation and the development of various tumours, including GI malignancies. This review presents general characteristics and the involvement of selected CLDNs in the progression of GI malignancies, with a focus on the potential application of these proteins in the diagnosis and prognosis of colorectal cancer (CRC), gastric cancer (GC), pancreatic cancer (PC), and oesophageal cancer (EC). Our review indicates that selected CLDNs, particularly CLDN1, 2, 4, 7, and 18, play a significant role in the development of GI tumours and in patient prognosis. Furthermore, selected CLDNs may be of value in the design of therapeutic strategies for the treatment of recurrent tumours.

## 1. Claudins (CLDNs)—General Information

**Figure 1:**
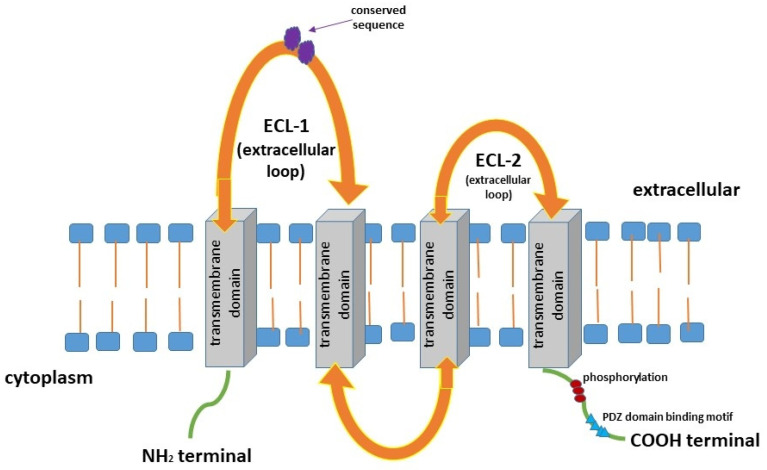
Structure of claudins (CLDNs) [1,2,3,4,5,17,18,19,25].

Claudins (CLDNs) belong to a family of at least 27 transmembrane proteins that range in size from 20 to 34 kDa. According to their degree of sequence similarity, these proteins were divided into two groups, classic claudins (CLDNs1–10, CLDN14, CLDN15, CLDN17, and CLDN19) and non-classic claudins (CLDNs11–13, CLDN16, CLDN18, and CLDNs20–24) [1,2,3,4,5]. In addition, this group of proteins can be functionally divided into barrier-forming and pore-forming claudins [2]. Pore-forming claudins such as CLDN2, -10b, and -15 are qualified as cation pores, while CLDN-10a and -17 are classified as anion pores [6,7,8,9,10,11,12,13]. Others CLDNs have been indicated to form pores only when specifically interacting with another claudin. Authors conclude that a combination of CLDN16/-19 has been reported to act as a cation pore and CLDN4/-8 as an anion pore [14,15]. CLDNs are encoded by a multi-gene family. Several pairs of highly homologous CLDN genes are located in close proximity in the human genome, e.g., *CLDN8* and *CLDN17* on chromosome 21, *CLDN3* and *CLDN4* on chromosome 7, *CLDN22* and *CLDN24* on chromosome 4, and *CLDN6* and *CLDN9* on chromosome 16 [1]. Claudins are composed of the N-terminal region of the cytoplasm, four transmembrane domains, two extracellular loops, and the C-terminal tail of the cytoplasm [16] (Figure 1). This family of proteins shares a structural topology of four putative transmembrane (TM) segments, a large extracellular loop of CLDNs that contains a consensus sequence motif, and a second shorter extracellular loop [1,2,3,4,5,6]. Furthermore, CLDNs create charge-selective channels that are able to regulate the paracellular ion selectivity while the second, shorter, extracellular loop presents a helix-turn-helix motif. In the structure of CLDNs, the first extracellular loop determines the ion selectivity of the CLDNs such as anion- or cation-selective channels and sealing. It was shown that several CLDNs, including CLDN1, -3, -5, -11, and -19, contain a non-charged first loop; therefore, they do not exhibit ion selectivity, and these proteins function to seal intercellular junctions [5,6,7,8,9,10,11,12,13]. Moreover, two cysteines of CLDNs form internal disulfide bonds, crucial in the stabilisation of protein conformation [16]. It has been proven that the C-terminal tail of the CLDNs exhibits diversity in sequence and length, and contains a PDZ-domain-binding motif, thus CLDNs are able to interact directly with cytoplasmic tight junctions (TJs). Post-translational modifications such as the phosphorylation of the tail region affect the localisation and functions of these proteins. It has been indicated that the phosphorylation of the C-terminal tail of CLDNs interacts with several major kinases and may downregulate TJ expression [1,16]. The study of Suzuki et al. has revealed that TM segments of mammalian claudin 15 (mCLDN15) form a typical left-handed four-helix bundle. Furthermore, large portions of the two extracellular segments form a characteristic β-sheet fold comprising two extracellular segments. These findings indicated that the linear alignment of mCLDN15 protomers discovered in the lipidic cubic phase (LCP) crystals could be representative of linear CLDN polymers, which are presented in TJs. Authors conclude that the idea of the crystal structure of CLDNs, a building block of TJ strands, will improve the molecular understanding of paracellular barriers between epithelial cells [15]. Claudins are major components of TJs which regulate the maintenance of epithelial cell polarity as well as the paracellular permeability [1]. Moreover, TJs are composed of transmembrane and peripheral membrane proteins involved in complex protein–protein interactions. TJs contain endogenous transmembrane proteins, including CLDNs and occludin, as well as cytoplasmic zonula occludens proteins that link the actin cytoskeleton and signalling proteins [17].

The main functions of the majority of CLDN family members are paracellular barrier formation, intramembranous diffusion barrier formation, and cell polarity [18,19,20]. These proteins are mainly localised in the apical region of the cell membrane and form a TJ complex for the maintenance of cell polarity and cell–cell adhesion. Therefore, it has been suggested that CLDNs play an important role in the functioning of the intercellular barrier formed by TJs [20].

CLDNs are expressed in a tissue-specific combination. The CLDN1 tissue expression has been observed in tight epithelia and human ovarian and brain epithelium; CLDN2 has been observed in leaky epithelia, e.g., intestinal crypts; while CLDN3 has been observed in the human gallbladder, brain capillary endothelium, and liver/intestinal epithelial cells. The tissue expression of CLDN6 was indicated in neonatal proximal tubules, whereas CLDN7, 8, 11, and 12 were indicated in the duodenum, jejunum, ileum, and colon thick ascending limb [21,22,23,24].

### 1.1. Claudins—Their Role in Tumour Pathogenesis

One of the hallmarks of cancer is a loss of cellular structure. The function of TJ proteins in the maintenance of normal glandular epithelial physiology has been widely investigated. However, the role of these proteins in the neoplastic process has not been fully elucidated. Most of the studies have examined the expression pattern of CLDNs in human malignancies using immunohistochemistry, but little is known about serum concentrations of CLDNs in patients with gastrointestinal (GI) cancers [26,27].

The cellular organisation characteristic of normal differentiated tissue is often lost in malignancy because cancer cells frequently exhibit decreased differentiation and cell polarity. This process is crucial to the development of an invasive phenotype and, consequently, metastasis [26,27]. It has been demonstrated that the first step in the metastasis of neoplastic cells is infiltration into the normal tissue as well as surrounding tumour-associated stroma. Epithelial–mesenchymal transition (EMT) is a crucial process for cancer cells to acquire a metastatic phenotype in mesenchymal cells [18]. EMT begins from the breakdown of adhesion between epithelial cells, such as TJs, adherens junctions, desmosomes, and gap junctions, which may cause a loss of cell polarity and cytoskeletal reorganisation. It has been proven that CLDNs as TJ proteins participate in all steps of cancer development [16,28].

**Figure 2:**
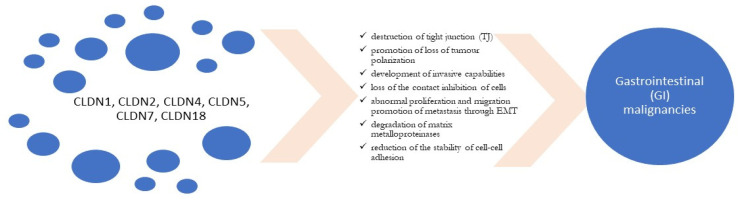
Role of claudins (CLDNs) in tumour development [31,32,33,34,35].

A growing body of evidence indicates that tumour growth is driven not only by tumour-cell-intrinsic mechanisms but may also be dependent on paracrine signals, including selected growth factors produced by the tumour microenvironment (TME). These molecules are synthesised as transmembrane proteins and are released by limited proteolysis defined as ectodomain shedding. Some clinical investigations have reported that the loss of the cell–cell adhesion complex is associated with increased EMT in cancer, while the phosphorylation and decreased expression of selected CLDNs may promote the infiltration and metastasis of tumour cells [29] (Figure 2). The overexpression of CLDNs in many malignancies, including GI cancers, may stimulate the promotion, progression, and metastasis of malignant cells [30]. The authors concluded that CLDNs are generally highly expressed in cancer tissues, although expression intensity depends on the location of the tumour and the type of CLDNs. This could be explained by a reduction or loss of function of CLDNs due to phosphorylation that may promote EMT as well as cancer infiltration and metastasis. Increased expression of CLDN1 has been observed in melanoma cells, while downregulation of this protein has been observed in squamous cell carcinoma, pancreatic cancer, and glioblastoma. Moreover, CLDN3 and CLDN4 were overexpressed in ovarian cancer, prostate cancer, breast cancer, and colorectal cancer, and CLDN5 was overexpressed in pancreatic cancer. Downregulation of CLDN7 was determined in breast cancer and head and neck cancer, whereas elevated levels of this protein were indicated in stomach cancer. Moreover, expression of CLDN18 was reduced in gastric cancer [15,17]. These results indicate the importance of various CLDNs in the pathogenesis of GI malignancies [29].

### 1.2. Selected Claudins—Their Role in Gastrointestinal Cancers’ (GI) Development and Prognosis

The loss of function of TJ proteins has been observed in several GI malignancies including colorectal, gastric, pancreatic, and oesophageal cancer [26,36]. The expression of selected CLDNs is altered in various types of cancer compared with normal tissue. The changes in CLDNs’ expression in tumour development have been demonstrated at transcriptional and post-transcriptional levels [37]. Transcriptional regulation by transcription factors, as well as epigenetic mechanisms, including DNA methylation, histone modification, or microRNAs (miRNAs), regulate the expression of CLDNs [38]. The authors suggest that these changes in CLDNs’ expression increase the motility and promote metastasis of cancer cells [27,39]. Some clinical investigations have demonstrated that selected CLDNs play a dual role in tumourigenesis. These proteins play a cancer-promoting or cancer-suppressing role in a tissue-dependent manner. Furthermore, their utility as prognostic factors and therapeutic targets has been evaluated [40,41].

## 2. Gastrointestinal Cancers (GI)—General Characteristics

Among gastrointestinal (GI) malignancies, colorectal (CRC) and gastric (GC) cancers are most common malignancies worldwide [42]. It is estimated that CRC is one of the most commonly diagnosed cancers and the second leading cause of cancer-related deaths worldwide. Gastric cancer is the fourth most common cancer worldwide that accounts for around 10% of all cancer-related deaths [43]. It is estimated that pancreatic cancer (PC), the incidence of which continues to increase yearly, is the seventh leading cause of cancer-related deaths worldwide due to its high invasiveness, frequent metastasis, and high recurrence rate [44].

Rapid progression and unfavourable prognosis are the principal features of tumours of the alimentary tract. The 5-year survival rate in PC patients is more the 12% [45]. The standard diagnosis of GI tumours involves endoscopic methods as well as computed tomography (CT), positron emission tomography–CT (PET-CT), and endoscopic ultrasound (EUS) [46]. Furthermore, an upper gastrointestinal endoscopy and biopsy are still crucial to confirm the diagnosis. Moreover, measurements of the classic serum tumour markers are also used in routine clinical practice. The assessment of the well-investigated classic tumour markers for GI malignancies, such as carbohydrate antigen 19.9 (CA 19.9) and 72.4 (CA 72.4) as well as carcinoembryonic antigen (CEA), is helpful in monitoring cancer progression but not useful in the early detection of these malignancies due to the insufficient diagnostic sensitivity and specificity offered by the above markers. A great challenge for future diagnostics is to discover new cancer-specific and organ-specific biomarkers that will allow for cancer to be accurately detected and diagnosed at an early stage, thus improving patient outcomes.

A number of authors have recently revealed that TJ proteins, in particular selected CLDNs, are associated with inflammation and the development of various tumours, including GI malignancies [1]. The overexpression of CLDN7 promotes proliferation, migration, invasive potential, and tumourigenesis of CRC, while decreased CLDN1 expression was proved to stimulate tumour cell invasion and metastases of PC. In addition, the elevated expression of CLDN11 inhibits the development of GC [41]. Moreover, CLDN7 and CLDN11 play cancer-promoting roles in the pathogenesis of CRC, whereas CLDN6 plays a cancer-promoting role in the pathogenesis of GC [1]. A tumour-suppressive function of CLDN1, 4, and 11 has been indicated for GC, while this function of CLDN4 has been indicated in the pathogenesis of PC [1]. The significance of selected claudins in GI malignancies is presented in Table 1.

Based on the most recent findings, we believe that selected CLDNs might be considered promising candidates for early biochemical indicators involved in GI carcinogenesis. Therefore, this review aims to demonstrate the significance of selected CLDNs, particularly CLDN1, 2, 3, 4, 7, and 18, in the development and metastasis of GI cancers as well as to present the potential application of these proteins in the diagnosis and prognosis of GI malignancies.

### 2.1. Colorectal Cancer

The morbidity and mortality of CRC are ranked third and fourth among malignant tumours, respectively [68,69]. It is estimated that around 80% of colorectal carcinomas are histologically well-to-moderately differentiated [29,30,36]. Some clinical investigations have reported an upregulated expression of selected CLDNs in CRC tissue using immunohistochemistry (IHC) [36,70]. The authors demonstrated that the upregulation of these proteins in CRC patients is associated with TJ disorganisation as well as increased paracellular permeability. Selected CLDNs might become potential markers and therapeutic targets in this type of cancer [36].

#### 2.1.1. CLDN1 and CLDN7

Claudin-7 (CLDN7) is a member of the claudin family that constitutes cell–cell TJs. Abnormal CLDN7 expression is related to the occurrence and development of various GI malignancies, including CRC, via the destruction of TJs, loss of contact inhibition of cells, and abnormal proliferation and migration [41]. Darido et al. revealed that elevated CLDN7 expression in CRC cells may lead to a loss of cell polarity and an increase in β-catenin/Tcf-4 activity, thus promoting CRC cells’ proliferation [31]. The authors concluded that an enhanced expression of CLDN7 may contribute to tumour-forming ability in vivo [31]. Contradictory findings have been presented by other authors who, based on the IHC technique, demonstrated significantly lower CLDN7 staining intensity in CRC tissue in comparison to adjacent non-tumour tissue [25,51]. Furthermore, significant correlations have been found between CLDN7 expression, lymphovascular and neurotropic invasion, lymphocyte status, and tumour grade [25,51,52]. Moreover, it has been demonstrated that reduced CLDN7 mRNA levels in early CRC promote damage of the epithelial barrier in adenomas [52]. Other authors have used the immunosorbent assay (ELISA) to assess serum CLDN7 levels in CRC patients and found that they were significantly reduced in cancer patients in comparison to the control group. The concentrations of this protein were correlated with a high T stage and elevated concentrations of CEA, the classic tumour marker for CRC [48]. These results are consistent with the findings of another study which revealed that CLDN7 levels were significantly lower in the sera of CRC patients in comparison to healthy controls. Furthermore, the authors indicated that the expression of this protein was decreased in CRC tissue in comparison to normal colorectal tissue, and it was positively correlated with the degree of CRC tissue differentiation assessed using IHC [71]. Moreover, CLDN7 expression levels were significantly reduced or undetectable in metastatic tissue compared with primary tumour tissue [71]. The authors concluded that CLDN7 downregulation is associated with CRC and metastasis, and it may be used as an early diagnostic marker and a novel therapeutic target [71]. The presented findings confirm that CLDN7 may be of significance as a predictive and prognostic biomarker for CRC [48,71].

Among CLDNs, CLDN1 possesses the ability to promote the invasive capabilities by tumour cells. Low CLDN1 expression is associated with advanced-stage tumours and poor prognosis in CRC patients, while no correlations have been found between the immunoreactivity of this protein and gender, tumour differentiation, depth of invasion, and lymph node involvement [32]. Furthermore, it has been indicated that reduced CLDN1 expression is an independent predictor of tumour recurrence associated with poor patient outcomes. In addition, the authors conclude that decreased CLDN1 expression is a strong predictor of disease recurrence and an unfavourable outcome in patients with stage II colon cancer [47]. Moreover, a study by Karabulat et al. demonstrated that serum levels of CLDN1 in CRC patients were significantly reduced in individuals with metastatic and non-metastatic CRC in comparison to healthy controls [48]. The authors also observed a correlation between CLDN1 levels in patients with non-metastatic and metastatic CRC. In addition, lower serum CLDN1 levels were significantly correlated with elevated CEA levels. However, the investigators failed to assess statistical relationships between serum CLDN1 levels and progression-free as well as the overall survival rates for patients with CRC [48].

#### 2.1.2. CLDN2

Claudin-2 (CLDN2), a well-investigated component of cellular TJs, has been indicated to be associated with CRC progression [50]. A study by Tabaries et al. demonstrated that the overexpression of this protein is associated with poor overall and liver metastasis-free survival. Furthermore, immunohistochemical analysis revealed elevated CLDN2 levels in replacement-type metastases in comparison to those with desmoplastic features, while CLDN8 was highly expressed in desmoplastic CRC liver metastases [49]. The authors concluded that CLDN2 status in patient-derived extracellular vesicles may serve as a relevant prognostic biomarker to predict the risk of replacement-type liver metastases’ development [49]. Moreover, CLDN2 could be used as a valuable tool in designing optimal treatment strategies to better manage CRC patients with liver metastases [49]. Furthermore, a study by Wei et al. also demonstrated that CLDN2 is upregulated in CRC. The expression of this protein is significantly elevated in CRC patients and is correlated with tumour metastasis and patient survival. The authors concluded that CLDN2 plays an oncogenic role in CRC progression because the overexpression of this protein is associated with poor survival of CRC patients, and it may serve as a promising target for the treatment of patients with this malignancy [50].

#### 2.1.3. CLDN18

Claudin-18 (CLDN18) plays a significant role in the formation of TJs, and the altered expression of this protein has been observed in various GI malignancies, including CRC. Matsuda et al. assessed the importance of CLDN18 in the pathogenesis of CRC using immunohistochemical analysis [53]. The authors found that the expression of CLDN18 as well as tumour size and the presence of distant metastases are independent predictors of the survival of patients with PC. Furthermore, positive CLDN18 expression in CRC is correlated with worse patient outcomes and is an independent predictor of the survival of CRC patients. The authors concluded that this protein may be a useful marker to predict poor prognosis in CRC patients [53].

#### 2.1.4. CLDN4

Claudin-4 (CLDN4) is a TJ protein that has also been indicated to play a role in the development of CRC. Ueda et al. evaluated the importance of this molecule in CRC pathogenesis [33]. Reduced CLDN4 expression was detected in 57% of patients with CRC, in particular in metastatic lesions and lesions of invasive potential. Furthermore, decreased CLDN4 expression was significantly correlated with depth of invasion, lymphatic vessel invasion, venous vessel infiltration, as well as the presence of lymph node, liver, and other distant metastases. These results indicated that changes in CLDN4 expression may enhance cancer cell invasion and metastasis in CRC. Therefore, CLDN4 might be a good biomarker for predicting the risk of distant metastasis [33].

### 2.2. Gastric Cancer

Despite recent improvements in the diagnosis of gastric cancer (GC) and the treatment of patients with this malignancy, this type of GI tumour is still characterised by a high recurrence rate and metastasis and poor patient outcomes. Therefore, the search for novel therapeutic targets associated with EMT and cell–cell adhesion to improve the treatment of GC patients continues [72,73].

#### 2.2.1. CLDN1 and CLDN2

It has been indicated that CLDN1 and CLDN2 may be involved in GC pathogenesis. CLDN1 expression is significantly correlated with the differentiation of GC, and the immunoreactivity of this protein is the highest in well and moderately differentiated GC cells [74]. The authors found that the expression of this protein was significantly related to the intestinal type by Lauren’s classification and higher histological grades of GC. Based on their findings, the authors concluded that CLDN1 is a potential biomarker for GC. No significant relationships between the expression of this claudin and vascular invasion, tumour stage or lymph node metastasis were demonstrated. The presented findings suggest that CLDN1 may serve as an indicator of GC differentiation [75]. Furthermore, CLDN1 overexpression is correlated with the presence of distant metastases, tumour infiltration, and worse prognosis [54,55].

Some clinical investigations have revealed that the expression of CLDN2 is higher in GC tissue in comparison to normal tissue [76,77]. Contradictory results have been presented by other authors who revealed that CLDN2 expression was lower in GC compared with adjacent normal mucosa [78]. Furthermore, the authors did not establish any significant relationships between CLDN2 expression and clinicopathological factors [78].

#### 2.2.2. CLDN4

Claudin-4 (CLDN4) is an integral membrane protein, the expression of which is frequently altered in various tumour tissues, including GC. A study by Zhu revealed that CLDN4 was expressed in 90.5% of intestinal metaplastic lesions and in 95.2% of dysplastic lesions, while it was present only in 15.9% of normal gastric tissue samples. Moreover, there was a significant relationship between CLDN4 expression and histological differentiation as well as tumour growth patterns. However, no correlations were found between the expression of this protein and patient survival [56]. The authors concluded that the expression of CLDN4 might serve as a biomarker for GC precursor lesions [56]. Furthermore, a meta-analysis has indicated that the expression of this claudin is correlated with increased tumour size and lymph node metastases in GC patients [79]. The authors concluded that altered levels of this protein may promote metastasis through the promotion of EMT [79]. In addition, Hwang et al. indicated that the overexpression of CLDN4 degrades extracellular matrix components and ultimately promotes the infiltration and motility of cancer cell metalloproteases, such as matrix metallopoteinase-2 (MMP-2) and metalloprotease-9 (MMP-9) [34]. It has been proven that CLDN4 is involved in the formation of TJs. Abnormal CLDN4 expression reduces the stability of cell–cell adhesion and promotes cancer development. Furthermore, there is a correlation between the increased immunoreactivity of CLDN4 and clinicopathological factors as well as inhibited GC cell infiltration and migration [29]. The presented results indicate the importance of CLDN4 in the pathogenesis of GC.

#### 2.2.3. CLDN3, CLDN7, and CLDN18

Some investigators have proven that CLDN3 expression is lower in GC patients with advanced tumour depth and positive lymphatic invasion, which confirms the role of this protein in GC progression [35,80,81]. A study by Jun et al. demonstrated that CLDN7 expression was significantly elevated in patients with the intestinal type of GC based on Lauren’s classification and in patients without nodal involvement. The decreased expression of CLDN18 was significantly correlated with perineural invasion [35]. The authors concluded that the upregulation of CLDN7 as well as the downregulation of CLDN18 play a role in the pathogenesis of GC. CLDN7 expression and loss of CLDN18 are independent indicators of poor prognosis in GC patients [35]. Some investigators have reported promising results from clinical trials that evaluated the usefulness of an anti-CLDN18.2 antibody as a therapeutic drug for GC [72,73].

#### 2.2.4. CLDN10, 14, 17, and 23

It has been demonstrated with the use of IHC that CLDN14 expression is higher in GC tissue when compared with adjacent normal mucosa and is correlated with lymph node metastasis [82]. Furthermore, the authors revealed that CLDN17 expression was reduced in GC tissue when compared with normal tissue. Additionally, a decreased expression of this claudin was positively correlated with the presence of lymph node metastasis [82].

A disruption of CLDN23 immunoreactivity has also been observed in GC patients. Lu et al. found that the expression of this claudin was lower in GC tissue in comparison to normal gastric mucosa. No significant differences were found between CLDN23 levels and lymph node metastasis or TNM stage. However, the overall survival of patients with GC was longer for those with negative CLDN23 expression, which was demonstrated using multivariate survival analysis [57]. Some evidence suggests that selected CLDNs might be potential biomarkers for GC progression and prognosis.

### 2.3. Pancreatic Cancer

The incidence of PC continues to increase yearly [44]. Due to a lack of characteristic symptoms and accurate screening methods, this malignancy is usually detected at an advanced stage. Therefore, new techniques and biomarkers are critically needed to improve the diagnosis of PC. Some clinical investigations have suggested the significance of selected CLDNs in the pathogenesis of PC. These proteins are members of TJs and participate in all steps of tumour development [72]. Moreover, the diagnostic utility of these proteins has also been reported. Some authors have indicated that CLDNs might be used in the treatment of PC patients via targeting different CLDNs and combining chemotherapy, stimulating tumour cell necrosis and inhibiting tumour invasion and metastasis [72]. However, CLDNs, similarly to matrix metalloproteinases (MMPs), play a dual role in tumour development. These proteins either promote or inhibit the development of cancer and, therefore, the specific biological properties of particular CLDNs need to be taken into consideration in the diagnostic process and in the design of treatment strategies [72].

#### 2.3.1. CLDN1 and CLDN4

Some studies have demonstrated that CLDN1 is frequently upregulated in PC and enhanced in metastatic tissue [1]. In addition, the expression of this protein is regulated by various growth factors as well as tumour necrosis factor (TNF-a) via concentration dependence. Decreased CLDN1 expression may promote the proliferation, invasion, and metastasis of tumour cells, including PC [58].

The overexpression of CLDN4 is correlated with the presence of liver metastases from PC [83] as well as with the histological grade of the tumour [84]. It has been indicated that PC patients with elevated CLDN4 expression have significantly longer survival times in comparison to patients with low tissue levels of this protein. The authors concluded that increased CLDN4 expression may predict better prognosis in PC [59]. Furthermore, some authors believe that CLDN4 may serve as a novel diagnostic biomarker for PC. A study by Tsutsumi et al. demonstrated that this molecule was negatively expressed in reactive mesothelial cells and mesothelioma, while it was significantly positively expressed in plasma membrane metastases and the primary carcinoma of PC [60]. The authors used qRT-PCR (quantitative real-time polymerase chain reaction) to assess the increased expression of CLDN4 in all PC cell lines tested compared with normal ductal epithelial cells and fibroblasts. Furthermore, reduced immunoreactivity of this claudin was significantly correlated with decreased patient survival. Moreover, in IHC analysis, the level of CLDN4 mRNA expression was significantly correlated with the expression of CLDN4. The investigators confirmed that the overexpression of CLDN4 mRNA predicts a better prognosis in PC [60]. Contrary to the above findings, some clinical investigations have reported that CLDN4 is an effective inhibitor of the invasive and metastatic phenotype of PC cells [84,85,86], which confirms the dual role of CLDN4 in the development of this malignancy [72].

#### 2.3.2. CLDN5 and CLDN7

CLDN5 plays an important role in maintaining the integrity of the blood–brain barrier. This protein has no paracellular pore function, thus it may serve as a marker for the endothelium [87]. It has been proven that CLDN5 is significantly positively expressed in cell membranes of the solid pseudopapillary tumour of the pancreas [87]. A study by Soini revealed that CLDN5 expression was correlated with worse patient survival, although they failed to demonstrate any significant relationships between CLDN5 immunoreactivity and tumour size or spread. In addition, the expression of this claudin was associated with enhanced apoptosis and an increased expression of the bax protein [88]. The presented results indicate the significance of CLDN5 in PC pathogenesis.

CLDN7 is expressed in normal and malignant pancreatic tissue, but its expression is decreased in pancreatic ductal adenocarcinoma and is correlated with the degree of PC differentiation. This protein regulates cell proliferation via integrins and maintains epithelial cell attachment, controlling the growth and cycle progression of cells and regulating tumour cell proliferation [89]. It has also been demonstrated that CLDN7 may be overexpressed in rapidly proliferating cell populations in PC tissues and could be used as a novel molecular target for PC treatment, similarly to CLDN12 and CLDN23 [61]. Some clinical investigations have proven that CLDN12 plays an oncogenic role in PC cells, promoting its malignant phenotype, and it might be a therapeutic target for inhibiting PC cell progression [90,91].

#### 2.3.3. CLDN18

CLDN18 is reported to have two specific isoforms (CLDN18.1 and CLDN18.2) that are present in lung and gastric tissue. CLDN18.1 is mainly expressed in normal lung tissue, whereas CLDN18.2 is restricted to gastric tissue, but it is also expressed in cell membranes under pathological conditions in cancerous tissue, such as GC, PC, and EC [92]. It has been demonstrated that CLDN18.2 might be aberrantly activated during the malignant transformation of pancreatic cells [93]. These findings indicate that this protein could be used as a promising biomarker for pancreatic ductal adenocarcinoma [62]. CLDN18.2 is expressed in primary as well as metastatic foci [63,64,72]. Therefore, this claudin is of significance in both patients with advanced PC and those who will develop metastatic PC. The authors revealed that the expression of CLDN18.2 was significantly increased in advanced-stage PC patients in comparison to early-stage patients. It has been demonstrated that enhanced CLDN18.2 expression is an unfavourable prognostic factor for lymph node metastasis. Furthermore, there is a significant positive correlation between the expression of this molecule and lymphatic infiltration as well as nerve invasion. It has been observed that CLDN18.2 expression is a statistically significant indicator of patient survival and prognosis. A worse prognosis has been revealed for CLDN18.2-positive patients with advanced PC with distant metastases [63,64,72].

### 2.4. Oesophageal Cancer

Oesophageal cancer (including squamous cell carcinoma and adenocarcinoma) is the eighth most commonly diagnosed cancer globally [94]. A rapid increase in the incidence of oesophageal adenocarcinoma (OAC) has been observed, particularly in the Western world. In the United States, the number of new OAC cases has increased 700% over the past decade [95]. A growing body of evidence indicates that selected CLDNs as TJ proteins might play an important role in the development and progression of oesophageal mucosal metaplasia, dysplasia, and carcinoma [95,96].

#### 2.4.1. CLDN1, CLDN2, and CLDN3

A study by Abu-Farsakh found that CLDN2 expression was significantly higher in OAC, squamous cell carcinoma (OSCC), and glandular lesions compared with squamous cell carcinoma, although no significant relationships were found between tissue levels of this protein and age, gender, grade, stage, or the survival time of patients with OAC and OSCC [65]. In addition, the authors indicated that gastroesophageal reflux disease may induce the overexpression of CLDN2 and increase the risk of the development of Barrett’s oesophagus. The presented study provides new insights into the role of TJ proteins such as CLDN2 in the pathogenesis of Barrett’s oesophagus and oesophageal cancer [65].

Gyõrffy et al. used IHC and mRNA expression analysis to assess the expression of selected claudins, including CLDN 1, 2, 3, 4, and 7 in different histological types of OC as well as in Barrett’s oesophagus and normal squamous epithelium [66]. CLDN 1 expression was significantly increased in OSCC compared with squamous epithelium. Moreover, the expression of CLDN3 and CLDN4 was significantly elevated both in Barrett’s oesophagus and OAC in comparison to the foveolar epithelium, while in OAC, the expression of CLDN2 was significantly higher in comparison to that in Barrett’s oesophagus. The authors suggest a link between Barrett’s oesophagus and OAC in selected CLDN patterns [66].

#### 2.4.2. CLDN4 and CLDN7

Usami et al. demonstrated that decreased CLDN7 expression in OSCC subjects was significantly associated with depth of invasion, clinicopathological stage of the tumour, lymphatic vessel invasion, and lymph node metastasis. However, the authors failed to observe any significant relationships between CLDN7 expression and the clinicopathological features of OC, suggesting that the downregulation of CLDN7 may lead to tumour progression and subsequent metastatic events [67]. Similar findings have been presented by other authors who also revealed that low CLDN4 expression was significantly associated with depth of invasion, lymph node metastasis, and histological differentiation. Furthermore, a reduced expression of this claudin has been found to have an unfavourable impact on disease-free survival and overall survival, and it has been proven to be an independent predictor of poor survival in OC patients, which was assessed using IHC and RT-PCR [97]. These findings prove that CLDN4 expression is deregulated in OSCC and might be a prognostic biomarker in this malignancy [97].

#### 2.4.3. CLDN18.2

A study by Moentenich et al. indicated that CLDN18.2 may serve as a promising therapeutic target in patients with OAC [67]. The authors used IHC to assess the expression of this molecule in OAC patients. No relationship was revealed between the expression of CLDN18.2 and clinicopathological characteristics, including tumour grade, tumour size and stage, and the presence of lymph node metastases. Furthermore, the study revealed the expression of this protein in a significant number of cases with lymph node metastases, highlighting the importance of CLDN18.2 in the development of novel targeted therapies for both histological types of OC and lymph node metastases [98].

## 3. Conclusions

Claudins (CLDNs) belong to a family of tetraspan transmembrane proteins of the TJs which form the paracellular barrier that regulates the flow of molecules in the intercellular space between the cells of the epithelium. Evidence of altered claudin levels in various human malignancies, including GI cancers, has been reported. GI cancers belong to the five most common malignancies worldwide and their incidence is still on the increase, particularly in Western countries. Therefore, there is an urgent need for more investigations into early biomarkers of these malignant diseases.

In this review, we present the involvement of selected claudins in the pathogenesis of the most common GI malignancies. Furthermore, the potential utility of these proteins in the diagnosis of GI cancers and patient prognosis has also been evaluated. This review demonstrates that among the analysed claudins, CLDN1 is able to promote the progression of CRC, GC, and PC as well as serve as a prognostic factor in patient survival. CLDN4 contributes to GC development and poor prognosis in GC and PC patients, and it may be a novel diagnostic biomarker for GC and PC. CLDN7 is a significant indicator of poor prognosis in GC patients and promotes progression of CRC, GC, and PC. Moreover, this protein may also be a novel molecular target in the treatment of PC patients. Furthermore, CLDN18 is correlated with worse patient prognosis in CRC, GC, and PC as well as being indicated as a biomarker for GC progression, while CLDN2 is demonstrated as a promising target in the treatment of CRC patients.

In conclusion, our review indicates that selected CLDNs, particularly CLDN1, 2, 4, 7, and 18, play a significant role in the development of GI tumours and in patient prognosis. In addition, selected CLDNs may be of value in the design of therapeutic strategies for the treatment of patients with these diseases. However, understanding of the biological significance of CLDNs is further clouded by the fact that, depending on the type of CLDN and tumour localisation, both their upregulation or downregulation are significantly related to the development and metastases of GI malignancies.

## Figures and Tables

**Table 1:** Clinical significance of selected claudins in GI malignancies.

Type of GI Malignancy	Claudins (CLDNs)	Significance	Material and Methods	References
Colorectal cancer (CRC)	CLDN1	✓ Low expression of CLDN1 is associated with advanced stage and poor prognosis ✓ Lower serum CLDN1 is significantly correlated with poor performance status	TM; IHC (tissue; 129 CRC patients) ELISA (serum, 140 CRC patients and 40 healthy controls)	[47] [48]
	CLDN2	✓ Overexpression of CLDN2 is associated with poor survival ✓ Promising target for the treatment of CRC patients	IHC (tissue) IHC (tissue, 104 CRC cases and 85 adjacent normal mucosa); cell culture (cell lines)	[49] [50]
	CLDN4	✓ Potential biomarker to assess the risk of distant metastasis	IHC (tissue, 129 cases and 44 metastatic tumours); siRNA (cultured cells)	[33]
	CLDN7	✓ Overexpression of CLDN7 might promote tumour progression ✓ Lower serum CLDN7 levels are significantly correlated with advanced T stage	IHC (tissue, 70 CRC cases) RT-PCR (tissue, 18 healthy individuals, 100 individuals with dysplasia and 121 CRC patients) ELISA (serum, 140 CRC patients and 40 healthy controls)	[25,51,52] [48]
	CLDN18	✓ Independent predictor of patient survival ✓ Potential biomarker to predict poor prognosis	IHC (tissue, 569 CRC cases)	[53]
Gastric cancer (GC)	CLDN1	✓ Overexpression of CLDN1 is correlated with tumour infiltration, metastasis, and poor survival	IHC (tissue, 173 GC cases); cell culture (cell lines); Western blotting (cell lysates) cDNA microarray analysis (20 patients)	[54] [55]
	CLDN3	✓ Biomarker for cancer progression	IHC (tissue, 134 GC cases)	[35]
	CLDN4	✓ Biomarker in precursor lesions ✓ Overexpression of CLDN4 is associated with clinicopathological factors and inhibits cell migration and infiltration	IHC (tissue, 329 GC cases, 44 normal stomach samples, 21 intestinal metaplasia samples, and 21 adjacent precursor lesions dysplasia samples)	[56]
	CLDN7	✓ Biomarker for cancer progression ✓ Independent indicator of poor prognosis	IHC (tissue, 134 GC cases)	[35]
	CLDN17	✓ Potential biomarker for cancer progression and prognosis	RNA extraction and RT-PCR, IHC (tissue, 109 GC cases)	[57]
	CLDN18	✓ Biomarker for cancer progression ✓ Independent indicator of poor prognosis	IHC (tissue, 134 GC cases)	[35]
	CLDN23	✓ Potential biomarker for cancer progression and poor prognosis	RNA extraction, RT-PCR, IHC (tissue, 109 GC cases)	[57]
Pancreatic cancer (PC)	CLDN1	✓ Decreased expression may promote proliferation, invasion, and metastasis of tumour cells	RT-PCR, siRNA, Western blot analysis (cell lines)	[58]
	CLDN4	✓ Overexpression of CLDN4 may predict better prognosis ✓ Novel diagnostic biomarker for PC	Western immunoblot, immunofluorescence (cells and tissue) qRT-PCR (mRNA expression, 9 cell lines), IHC (tissue, 20 PC cases)	[59] [60]
	CLDN7	✓ Novel molecular target for treatment of PC patients ✓ Biomarker for cancer progression	Cell culture (cell lines)	[61]
	CLDN18	✓ Biomarker for adenocarcinoma of pancreatic bile duct tumours ✓ Indicator of PC patient survival and prognosis	IHC (tissue), RT-PCR (cell lines) IHC (tissue, 93 PC cases, 86 para-cancer tissues, and 13 normal pancreatic tissue) IHC (tissue)	[62] [63] [64]
Oesophageal cancer (OC)	CLDN2	✓ Overexpression of CLDN2 increases the risk of the development of Barrett’s oesophagus	IHC (tissue, 111 OE cases) IHC (tissue, 125 OE cases)	[65] [66]
	CLDN18.2	✓ Novel targeted therapies for both types of OC and lymph node metastases	Western blot analysis, IHC (tissue)	[67]

IHC, Immunohistochemistry; siRNA, small interfering RNA; RT-PCR, quantitative real time polymerase chain reaction; TM, tissue microarray construction.

## Data Availability

The data can be shared up on request.
